# The role of VI-RADS scoring criteria for predicting oncological outcomes in bladder cancer

**DOI:** 10.1007/s00345-024-05101-2

**Published:** 2024-07-24

**Authors:** Mieszko Kozikowski, Magdalena Zagrodzka, Marek Zawadzki, Przemysław Zugaj, Rafał Osiecki, Franciszek Rzymkowski, Mateusz Śledź, Roman Sosnowski, Jakub Dobruch, Francesco Del Giudice, Wojciech Krajewski

**Affiliations:** 1 Polish Center of Advanced Urology, Department of Urology, St. Anne’s Hospital EMC, Piaseczno, Poland; 2 Department of Diagnostic Imaging - Quadia, Piaseczno, Poland; 3 Institute of Outcomes Research, Maria Sklodowska-Curie Medical Academy, Warsaw, Poland; 4 Section of Urologic Oncology of Polish Urological Association, Warsaw, Poland; 5 Department of Urology and Oncological Urology, Warmian-Masurian Cancer Center, Olsztyn, Poland; 6 Urology Clinic of Medical Postgraduate Education Centre, Department of Urology, Independent Public Hospital them. prof. W. Orlowski, Warsaw, Poland; 7 Department of Maternal Infant and Urologic Sciences, “Sapienza” University of Rome, Policlinico Umberto I Hospital, Rome, Italy; 8 Department of Minimally Invasive and Robotic Urology, University Center of Excellence in Urology, Wroclaw Medical University, Wroclaw, Poland

**Keywords:** Bladder cancer, Multiparametric magnetic resonance imaging, Recurrence, Bladder cancer staging

## Abstract

**Purpose:**

Our purpose was to evaluate the prognostic value of Vesical Imaging Reporting and Data System (VI-RADS) in bladder cancer (BCa) staging and predicting recurrence or progression.

**Methods:**

We retrospectively analyzed the prospectively collected data from 96 patients with bladder tumors who underwent VI-RADS-based multiparametric magnetic resonance imaging (mpMRI) before endourological treatment from April 2021 to December 2022. Diagnostic performance was evaluated by comparing mpMRI reports with final pathology, using logistic regression for muscle-invasive bladder cancer (MIBC) predictors. Follow-up until May 2023 included Kaplan-Meier and Cox regression analysis to assess VI-RADS predictive roles for recurrence-free survival (RFS) and progression-free survival (PFS).

**Results:**

A total of 96 patients (19.8% women, 80.2% men; median age 68.0 years) were included, with 71% having primary tumors and 29% recurrent BCa. Multiparametric MRI exhibited high sensitivity (92%) and specificity (79%) in predicting MIBC, showing no significant differences between primary and recurrent cancers (AUC: 0.96 vs. 0.92, *P* = .565). VI-RADS emerged as a key predictor for MIBC in both univariate (OR: 40.3, *P* < .001) and multivariate (OR: 54.6, *P* < .001) analyses. Primary tumors with VI-RADS ≥ 3 demonstrated significantly shorter RFS (*P* = .02) and PFS (*P* = .04).

**Conclusions:**

In conclusion, mpMRI with VI-RADS has a high diagnostic value in predicting MIBC in both primary and recurrent BCa. A VI-RADS threshold ≥ 3 is a strong predictor for MIBC, and in primary tumors predicts early recurrence and progression.

**Supplementary Information:**

The online version contains supplementary material available at 10.1007/s00345-024-05101-2.

## Introduction

Bladder cancer (BCa) ranks as the fourth most common malignancy in men and the fourth leading cause of cancer-related deaths in old age [1]. Accurate staging is crucial for therapeutic decisions in BCa patients, as non-muscle-invasive bladder cancer (NMIBC) carries a different prognosis and necessitates distinct treatment options compared to muscle-invasive bladder cancer (MIBC) [2]. NMIBC accounts for approximately 75% of BCa cases, posing a significant challenge in predicting disease recurrence and progression [3]. The risk of NMIBC progressing to MIBC within 5 years after primary transurethral resection of the bladder tumor (TURBT) varies widely, from less than 1% to over 40% among risk groups, influencing treatment choices [4].

Multiparametric magnetic resonance imaging (mpMRI) is increasingly used in clinical practice to enhance bladder tumor staging [5]. The recently introduced Vesical Imaging-Reporting and Data System (VI-RADS) has facilitated widespread adoption of mpMRI by offering standardized protocols and reporting criteria for bladder imaging [6]. Despite growing interest, there remain limited reports on its prospective use for predicting BCa stage and oncological outcomes [7, 8]. However, ongoing clinical trials challenging the standard BCa staging pathway show promising results [9]. To fully realize VI-RADS’s potential in BCa diagnosis, its impact on decision-making and predictive role for oncological outcomes must be demonstrated. Our study aimed to assess VI-RADS’s prognostic value in tumor staging and predicting BCa recurrence or progression.

## Materials and methods

### Patients and study design

The study was a retrospective analysis of prospectively collected data from single center of 96 patients with bladder tumors who underwent 1.5-Tesla mpMRI before TURBT. Between April 2021 and November 2022, 448 patients were referred for TURBT (Supplementary Information 1). The decision to perform mpMRI was at the discretion of the attending urologist. Eligible patients were 18 to 90 years old with primary tumors and recurrent NMIBC. Exclusions were due to severe renal impairment or allergies contraindicating contrast administration, contraindications to mpMRI (pacemaker, metal clips, etc.), lack of consent for standard surgical treatment related to BCa, previous treatment, or malignancies affecting bladder assessment in mpMRI. The study received institutional ethical board approval, and written informed consent was obtained from all enrolled patients.

The primary outcome measure was the diagnostic performance of mpMRi in differentiating between NMIBC and MIBC. As a secondary outcome measure, we planned an exploratory analysis examining the short- and long-term oncological outcomes, such as: the diagnostic performance of mpMRI in primary and recurrent BCa cases and in the case of NMIBC, recurrence-free survival (RFS) and progression-free survival (PFS) among patients stratified by VI-RADS. PFS and RFS were defined as the time to T stage extension from Ta/T1 or CIS to ≥ T2 or histopathologically confirmed recurrence, respectively.

### Multiparametric magnetic resonance imaging

mpMRI was conducted for staging before TURBT, and results were reported following VI-RADS guidelines [6]. Images were acquired using the Siemens Sola 1.5T MRI machine per the standardized VI-RADS protocol, which included: T2-weighted turbo spin echo images in axial, sagittal, and coronal planes; axial DWI images with high B value (b = 50–800 s/mm² and calculated b = 1500s/mm²); apparent diffusion coefficient maps were calculated for each plane; dynamic contrast-enhanced, fat-suppressed T1-weighted images were obtained in the axial plane with 5s temporal resolution. The images were interpreted independently by two radiologists, MZ (a specialist with 23 years of experience) and FR (a resident in-training), who were blinded to patients’ clinical information. Each lesion received a VI-RADS score of 1–5 based on the degree of muscularis layer invasion.

### Endoscopic treatment and histopathology

All patients underwent endoscopic treatment at our institution within a mean of 19 days (IQR: 2.75–30.25 days) after mpMRI. Procedures included bipolar TURBT or holmium/thulium laser en-bloc resection of bladder tumor (LERBT). Single intravesical installation was administered in selected cases. An expert uropathologist revised tumor samples according to the 1993 and 2004/2016 WHO classifications, describing grade, stage, presence of detrusor muscle, and completeness of resection in selected en-bloc cases (Table 1). Following EAU guidelines, incomplete primary TURBT prompted reTURBT, especially in cases lacking detrusor muscle in the specimen for > Ta G1/LG tumors and all T1 tumors [2]. Intermediate-risk and high-risk group patients received repeated intravesical chemotherapy installations or bacillus Calmette-Guérin immunotherapy, respectively, as adjuvant treatments. RFS and PFS of NMIBC cases were noted during the follow-up, which was terminated in May 2023. Patients with MIBC were excluded from the follow-up.

**Table 1:** Clinical characteristics and resection specification of the study group by primary tumors and recurrent cancers

	All	Primary tumor	Recurrent cancer	*P* Value
Variables *n* (%)	(*n* = 96)	(*n* = 68)	(*n* = 28)	
Age (median in years) [IQR]	68.0 [63.0-72.3]	68.0 [63.0–73.0]	66.0 [61.3–70.5]	0.27
Gender				0.87
female	19 (19.8)	13 (19.1)	6 (21.4)	
male	77 (80.2)	55 (80.9)	22 (78.6)	
Tumor diameter (median in mm) [IQR]	18 [9–30]	23 [13–30]	10 [5–17]	< 0.001
Multifocality				0.65
no	65 (67.7)	42 (61.8)	19 (67.9)	
yes	31 (32.3)	26 (38.2)	9 (32.1)	
mpMRI VI-RADS				0.05
VI-RADS 1	23 (24.0)	11 (16.2)	12 (42.8)	
VI-RADS 2	44 (45.8)	36 (52.9)	8 (28.6)	
VI-RADS 3	19 (19.8)	14 (20.6)	5 (17.9)	
VI-RADS 4	5 (5.2)	4 (5.9)	1 (3.6)	
VI-RADS 5	5 (5.2)	3 (4.4)	2 (7.1)	
Resection type				
TURBT	54 (56.3)	34 (50.0)	20 (71.4)	0.089
ERBT	42 (43.7)	34 (50.0)	8 (28.6)	
Energy modality				
bipolar	58 (60.4)	38 (55.9)	20 (71.4)	0.372
holmium laser	30 (31.3)	24 (35.3)	6 (21.4)	
thulium laser	8 (8.3)	6 (8.8)	2 (7.2)	
T stage				0.004
≤ Ta	29 (30.2)	16 (23.5)	13 (46.3)	
T1a	47 (48.9)	40 (58.8)	7 (25.0)	
T1b	6 (6.3)	5 (7.4)	1 (3.6)	
≥ T2	12 (12.5)	7 (10.3)	5 (17.9)	
Cis	2 (2.1)	0 (0.0)	2 (7.2)	
Grade WHO 1973				0.523
G1	28 (29.1)	20 (29.4)	8 (28.6)	
G2	62 (64.6)	45 (66.2)	17 (60.7)	
G3	6 (6.3)	3 (4.4)	3 (10.7)	
Grade WHO 2004/2016				0.599
low-grade	73 (76.0)	53 (77.9)	20 (71.4)	
high-grade	23 (24.0)	15 (22.1)	8 (28.6)	
Follow-up procedure				0.808
Cystoscopy	50 (52.1)	6 (8.8)	2 (7.2)	
reTURBT	30 (31.3)	33 (48.6)	17 (60.6)	
RC/TMT	8 (8.3)	23 (33.8)	7 (25.0)	
lost to follow-up	8 (8.3)	6 (8.8)	2 (7.2)	
Intravesical chemotherapy instillations				0.9529
HIVEC	33 (34.4)	24 (35.3)	9 (32.1)	
				< 0.001
BCG	6 (6.3)	0 (0.0)	6 (21.4)	

*Unless otherwise indicated, data are number of patients and data in parentheses are percentages. BCG = bacillus Calmette-Guérin, Cis = carcinoma in situ, ERBT = en bloc resection of bladder tumor, HIVEC = hyperthermic intravesical chemotherapy, IQR = interquartile range, mpMRI = multiparametric magnetic resonance imaging, RC = radical cystectomy, TMT = trimodality treatment, TURBT = transurethral resection of bladder tumor, VI-RADS = Vesical Imaging-Reporting and Data System, WHO = World Health Organisation

### Statistical analysis

Descriptive statistics were presented as numbers and percentages, or means ± standard deviations (SD), as appropriate. Diagnostic performance was evaluated using sensitivity, specificity, positive and negative predictive values, and accuracy, with 95% confidence intervals (CI). Receiver operating characteristic (ROC) curve analysis determined the area under the curve (AUC) for each subgroup. Data were analyzed using chi-squared test, Fisher’s exact test, DeLong test or the student’s t-test, as appropriate, with significance set at *P*-value < 0.05. The statistical analysis was conducted using the R program (version 3.2.2, R basis for statistical calculations, www.r-project.org) with appropriate packages. Univariate and multivariate logistic regression assessed the predictive impact of potential factors on MIBC, RFS, and PFS. RFS and PFS were calculated from the surgery date to the recurrence, progression, or last contact date. MK performed the statistical analysis.

## Results

Overall, 96 patients with 161 bladder tumors identified by mpMRI were included in this study (Supplementary Information 1). 68 (71%) had primary tumors and 28 (29%) were recurrent BCa cases. Clinical characteristics and surgical details of the study group divided into primary tumors and recurrent cancers are summarized in Table 1. Tumor diameter was larger in primary (23 mm, IQR: 13–30 mm) than in recurrent (10 mm, IQR: 5–17 mm) cases, which was the only significant preoperative clinical difference between these subgroups (*P* < .001). Understandably, in the group with recurrent cancers, BCG therapy was significantly more frequently administered (*P* < .001).

mpMRI exhibited high sensitivity (92%) and specificity (79%) in predicting MIBC across all patients (Supplementary Information 2), resulting in good diagnostic performance (AUC 0.93 [95%CI: 0.85-1.00]). The diagnostic value of mpMRI was comparable in primary and recurrent cases (AUC 0.96 [95%CI: 0.91-1.00] vs. 0.92 [95%CI: 0.79-1.00], *P* = .565), with slightly higher sensitivity (100% vs. 80%) and lower specificity (78% vs. 83%) observed in primary tumors compared to recurrent BCa. The optimal threshold for predicting MIBC was calculated as VI-RADS ≥ 3 in each group.

Patients underwent either TURBT in 54 (56.3%) or LERBT in 42 (43.7%) cases (Table 1). Energy modalities used during resection included bipolar in 58 (60.4%), holmium laser in 30 (31.3%), and thulium laser in 8 (8.3%) cases. Pathological examination indicated 84 (87.5%) NMIBC and 12 (12.5%) MIBC cases, with the following stages: 29 (30.2%) ≤ Ta, 47 (48.9%) T1a, 6 (6.3%) T1b, 12 (12.5%) T2, 2 (2.1%) Cis. In primary tumors, the most common stages were T1a diagnosed in 40 (58.8%) patients, followed by Ta in 16 (23.5%), and MIBC in 7 (10.3%) cases. In recurrent cases, the most prevalent stage was Ta in 13 (46.3%), followed by T1a in 7 (25.0%), and T2 stage in 5 (17.9%) patients, with postoperative stage being the main difference between subgroups (*P* = .004). BCa aggressiveness was defined as high-grade in 23 (24.0%) and low-grade in 73 (76.0%) tumors according to WHO 2004/2016 classification, and using the older 1973 grading, there were 28 (29.1%) G1, 62 (64.6%) G2, and 6 (6.3%) G3 cases without significant differences between subgroups (*P* = .599 and 0.523). The univariate and multivariable analysis of possible prognostic factors on MIBC are summarized in Supplementary Information 3. The VI-RADS score remained the single most important predictor of MIBC in both univariate (OR 40.3; *P* < .001) and multivariate analysis (OR 54.6; *P* < .001).

After excluding 12 patients with MIBC, who were scheduled for RC or TMT, and one refusing the proposed treatment, 83 patients with NMIBC were evaluated for RFS and PFS (Supplementary Information 1). In the followed-up patients following scores were assigned: VI-RADS 1 in 23 (27.7%), VI-RADS 2 in 42 (50.6%), VI-RADS 3 in 16 (19.3%), VI-RADS 4 in 2 (2.4%) patients, and none had VI-RADS 5 tumor. Median follow-up was 9.7 months (IQR: 5.4–12.7 months, range: 1.0-20.9 months). During that time the recurrence rate was 33.7%, while the progression rate was 8.4% in the group of NMIBC patients subjected to follow-up. Median RFS was 14.0 months (95%CI: 12.7-NA) and median PFS was not reached.

**Fig. 1:**
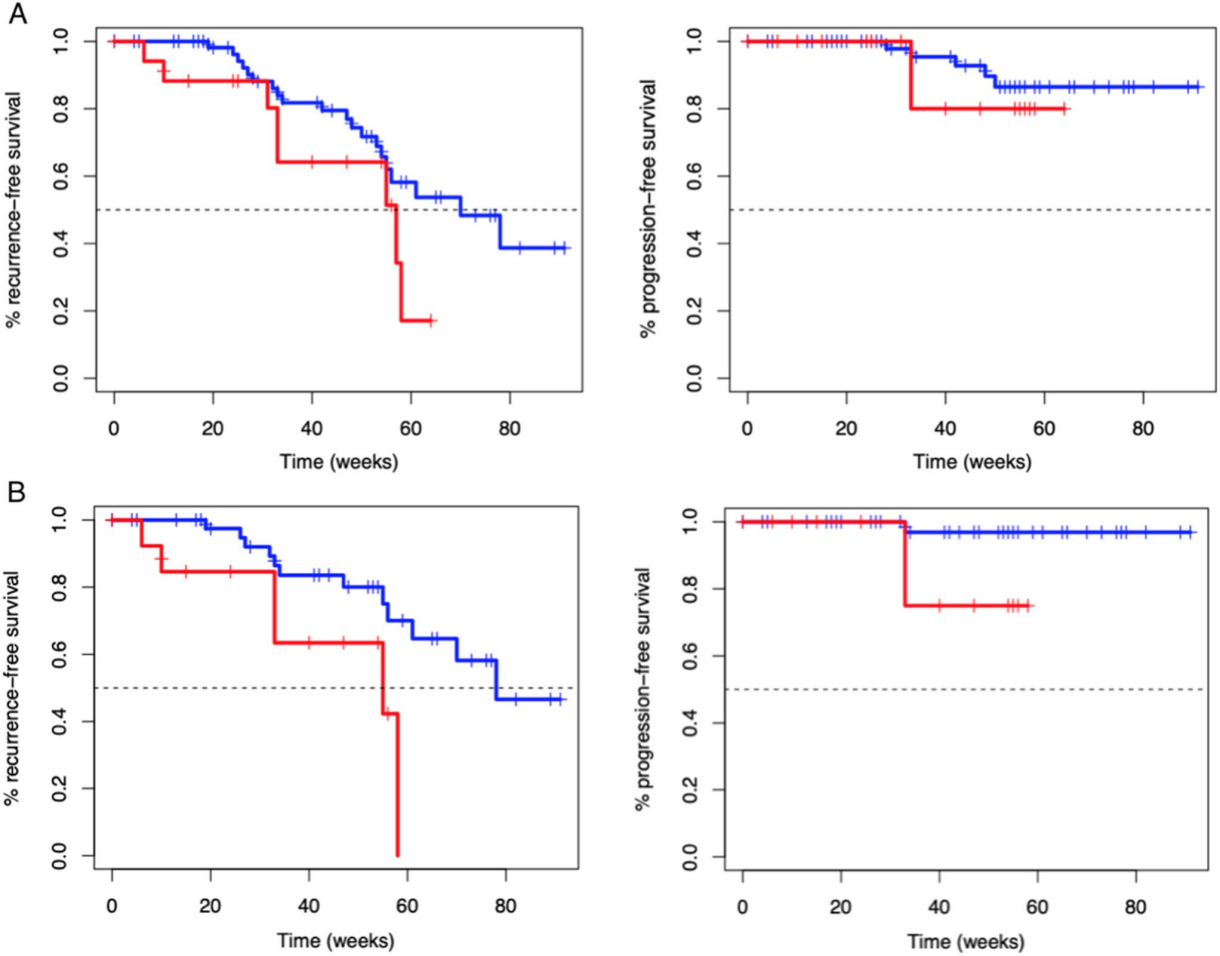
Kaplan-Meier survival curves for recurrence-and progression-free survival in the whole group of patients (**A**) and with primary tumors (**B**) stratified by VI-RADS ≤ 2 (blue) and VI-RADS ≥ 3 (red)

Patients were grouped into VI-RADS ≤ 2 (78.3%) and VI-RADS ≥ 3 (21.7%) cases. Median RFS in VI-RADS ≤ 2 group was 16.1 months (95%CI: 12.7-NA) and median RFS in VI-RADS ≥ 3 group was 13.1 months (95%CI: 7.6-NA), which difference was not significant in nonparametric (*P* = .1) and semiparametric analysis (HR: 1.96 [95%CI: 0.85–4.54], *P* = .1). Median PFS in patients with VI-RADS ≤ 2 and VI-RADS > 3 was not reached and the comparison did not show any difference in nonparametric (*P* = .5) and semiparametric analysis [HR: 1.82, 95%CI: 0.35–9.39, *P* = .5] in the total group (Fig. 1A).

In the analysis of the subgroup with primary tumors, patients with VI-RADS ≤ 2 had significantly longer median RFS (median 17.9 months [95%CI: 14.0-NA]) than with VI-RADS ≥ 3 tumors [median 12.7 months [95%CI 7.6-NA months], *P* = .02). Primary tumors assessed as VI-RADS ≥ 3 are over three times more likely to recur than VI-RADS ≤ 2 tumors (HR: 3.28 [95%CI: 1.15–9.31], *P* = .026) during the follow-up. All cases of VI-RADS ≥ 3 primary tumors relapsed before 14 months. However, median PFS for primary tumors was not reached, nonparametric analysis revealed a trend for shorter PFS in VI-RADS ≥ 3 primary tumors than in VI-RADS ≤ 2 primary tumors (*P* = .04, Fig. 1B), which was not confirmed in semiparametric analysis (HR: 8.67 [95%CI: 0.79–95.68], *P* = .078). No such relationships among recurrent cancers were found for RFS (*p* = .5) and PFS (*P* = .3).

## Discusion

mpMRI facilitates preoperative staging of BCa, crucial for determining the appropriate treatment. Our study affirms VI-RADS’ high diagnostic value in distinguishing NMIBC from MIBC under real-life conditions, despite a relatively low prevalence of ≥ T2 stage. Furthermore, VI-RADS maintains satisfactory diagnostic value for both primary tumors and recurrent BCa. To our knowledge, this is the inaugural study showcasing VI-RADS’ predictive role for recurrence and progression in primary tumors, potentially extending its applications beyond diagnostic accuracy.

Unlike many other malignancies, BCa has long lacked suitable imaging modalities for local staging. A primary challenge lies in acquiring adequate spatial resolution to discern bladder wall layers, crucial for assessing tumor infiltration depth [6]. Consequently, transurethral resection of the bladder tumor (TURBT) has remained the cornerstone staging tool for BCa [4]. Nonetheless, TURBT presents limitations including the need for surgical intervention with anesthesia, method inaccuracies, and the necessity for repeat procedures in high-risk cases prone to understaging. With the advent of VI-RADS, numerous studies have validated its high diagnostic accuracy in distinguishing between NMIBC and MIBC, propelling mpMRI as a promising pre-TURBT staging modality for BCa. However, a recent meta-analysis highlights that most of these reports are retrospective cohort studies [8]. Considering a VI-RADS threshold of ≥ 3 for predicting MIBC, the pooled sensitivity and specificity in the meta-analysis were 87% and 86%, respectively, comparable to our study findings (92% sensitivity, 79% specificity). The meta-analysis’s hierarchical summary ROC yielded an area under the curve of 0.93, mirroring the good diagnostic performance of mpMRI in our study, especially given the relatively low MIBC prevalence in our patient cohort (12%). Notably, only two studies in the cited meta-analysis featured patients with MIBC prevalence in the single-digit range, significantly impacting test diagnostic accuracy [10].

Postoperative inflammation or fibrosis can complicate MRI interpretation following endoscopic intervention [11]. Nevertheless, we observed no significant difference in mpMRI’s diagnostic efficacy between primary and recurrent tumors, the first such comparison to our knowledge. Despite stringent follow-up, recurrent bladder cancers detected were smaller and at lower stages than primaries, potentially impacting mpMRI sensitivity and specificity. The VI-RADS threshold of ≥ 3 for predicting MIBC increases false positives but enhances sensitivity, crucial in this context to avoid MIBC underdiagnosis [12]. Current NMIBC treatment standards advocate reTURBT in selected, mainly high-risk cases, yet its universal application remains debatable. While meta-analyses indicate no survival improvement with reTURBT in all T1 tumors, selected subgroups may benefit [13]. Our series exhibited a relatively high T1 tumor percentage (55.2%), likely due to frequent en bloc resection use (43.7%), which enhances T1 staging [14]. VI-RADS could aid patient selection for reTURBT, as suggested by prospective studies [7, 15]. Among potential MIBC prognostic factors assessed, VI-RADS score consistently emerged as the most significant predictor in both univariate (OR 40.3; *P* < .001) and multivariate (OR 54.6; *P* < .001) analyses.

To guide further treatment and follow-up scheduling post-TURBT, accurate prediction of disease recurrence and progression in NMIBC patients is paramount [2, 4]. Interestingly, none of the recognized factors sufficiently predicted recurrences or progression in the total group of patients, which may be due to the limited follow-up period in our study (Supplementary Information 4). Despite the VI-RADS scale being developed exclusively for assessing the stage of BCa, we chose to conduct an exploratory analysis to evaluate short- and long-term oncological outcomes in NMIBC patients stratified by VI-RADS. We discovered that a VI-RADS threshold of ≥ 3 accurately predicts shorter RFS (12.7 months vs. 17.9 months, *P* = .02) and possibly shorter PFS (median not reached, *P* = .04) in primary tumors. Notably, all VI-RADS ≥ 3 primary NMIBC cases recurred before 14 months, suggesting the need for more rigorous follow-up in these patients. Such correlations were not evident in the total patient group or in recurrent BCa. Our findings can be compared with a retrospective analysis investigating the inchworm sign’s presence in bladder mpMRI and its relation to outcomes post-TURBT [16]. The inchworm sign, indicating non-muscle invasive tumors forming a stalk, is considered a favorable prognostic factor [17]. It has indirectly been integrated into the VI-RADS system as one of the radiologic features characterizing VI-RADS 1 and 2 tumor categories. Nakagawa et al. found no significant differences in RFS (HR: 1.49 [95%CI: 0.98 − 2.27], *P* = .06) and PFS (HR: 1.92 [95%CI: 0.62 − 5.96], *P* = .28) between NMIBC groups with and without the inchworm sign. This underscores the rationale that a prognostic scale like VI-RADS, considering several structural and multiparametric characteristics, should be used instead of relying on a single radiologic feature. Recently, a nomogram incorporating various MRI features to predict MIBC in VI-RADS 2 tumors with a stalk demonstrated good diagnostic value (AUC 0.940) [18]. A growing body of literature suggests that automatic image analysis methods can further enhance bladder mpMRI interpretations [19].

Our study has some limitations. First, this was the single-center prospective study in which we did not assess interobserver agreement because this issue had already been addressed in the literature and controversial cases in our institution were resolved by consensus reading [12]. Second, the decision to perform mpMRI was left to the discretion of the attending physician, and recruiting consecutive patients would reduce possible selection bias, although it reflects real-world clinical practice. Finally, the number of study patients and follow-up time were insufficient to perform a complete survival analysis. Further studies with longer follow-up periods are required to accumulate more data.

In conclusion, our study confirms a high diagnostic value in predicting MIBC of mpMRI with VI-RADS in both primary and recurrent BCa. A VI-RADS threshold ≥ 3 may be a strong predictor for MIBC, and in primary tumors predicts early recurrence and progression.

## Electronic supplementary material

Below is the link to the electronic supplementary material.

Supplementary Material 1

Supplementary Material 2

Supplementary Material 3

Supplementary Material 4

Supplementary Material 5
