# Supplementary figures and images for: The role of VI-RADS scoring criteria for predicting oncological outcomes in bladder cancer
Source: World J Urol. 2024 Jul 24;42(1):438. doi: 10.1007/s00345-024-05101-2 (PMC11269435; doi:10.1007/s00345-024-05101-2)

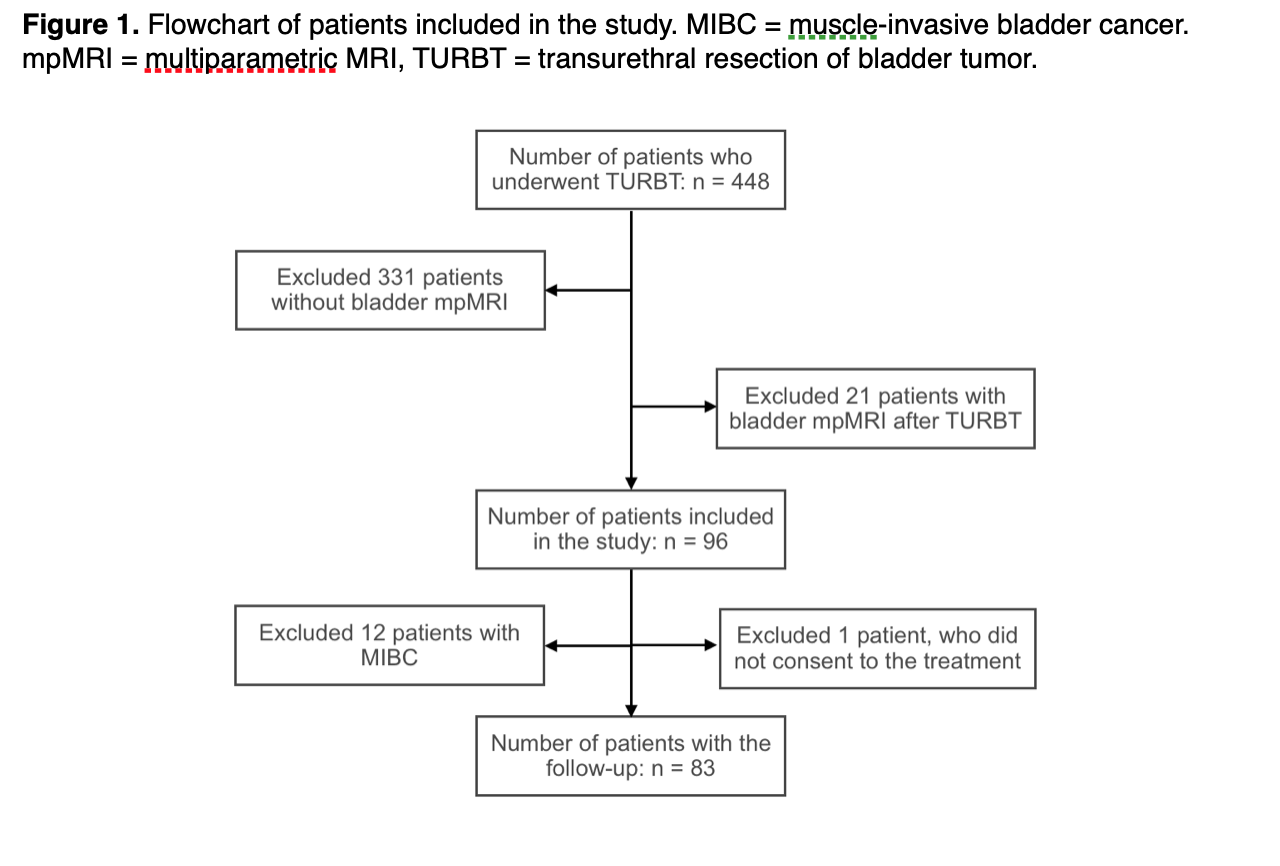

Supplement: Supplementary file 1 — Supplementary Material 1 [file 345_2024_5101_MOESM1_ESM.tiff]

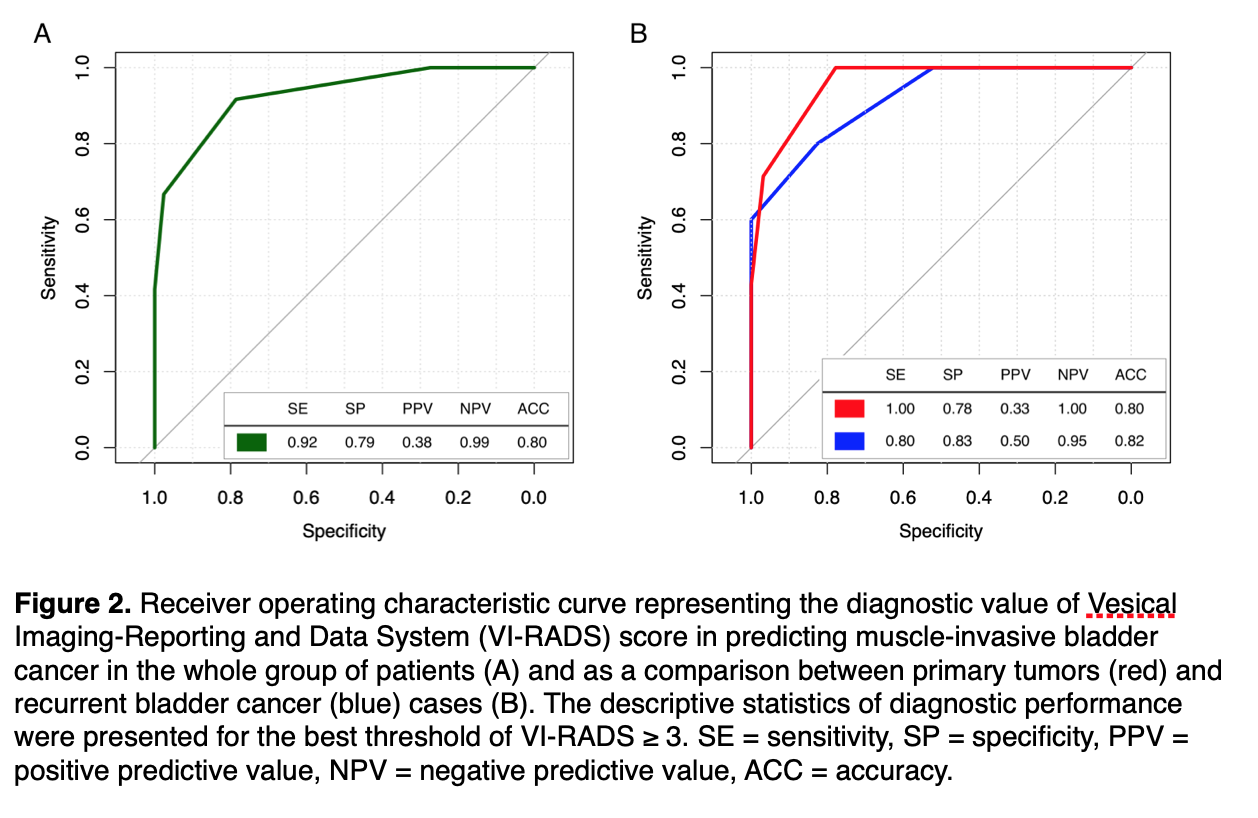

Supplement: Supplementary file 2 — Supplementary Material 2 [file 345_2024_5101_MOESM2_ESM.tiff]
