# Supplementary material for: The role of VI-RADS scoring criteria for predicting oncological outcomes in bladder cancer
Source: World J Urol. 2024 Jul 24;42(1):438. doi: 10.1007/s00345-024-05101-2 (PMC11269435; doi:10.1007/s00345-024-05101-2)
Supplement: Supplementary file 3 — Supplementary Material 3 [file 345_2024_5101_MOESM3_ESM.docx]

| **Supplementary Information 3. Univariate and multivariable analysis of the impact of possible prognostic factors of MIBC in the whole group.** | | | | | | | | | |
| --- | --- | --- | --- | --- | --- | --- | --- | --- | --- |
| Variables *N* (%) |  |  |  | univariate | | | multivariable | | |
|  | NMIBC | MIBC | *P* Value | OR | 95% CI | *P* Value | OR | 95% CI | *P* Value |
| Age |  |  | 0.357 | 1.80 | 0.52, 6.24 | 0.3 | 2.51 | 0.50, 14.7 | 0.3 |
| ≤ 70 years | 54 (56.2) | 6 (6.3) |  |  |  |  |  |  |  |
| > 70 years | 30 (31.2) | 6 (6.3) |  |  |  |  |  |  |  |
| Gender |  |  | 0.45 | 3.00 | 0.53, 56.6 | 0.3 | 6.78 | 0.68, 182 | 0.2 |
| female | 18 (18.8) | 1 (1.0) |  |  |  |  |  |  |  |
| male | 66 (68.8) | 11 (11.4) |  |  |  |  |  |  |  |
| Tumor diameter |  |  | 0.289 | 2.29 | 0.62, 7.97 | 0.2 | 0.75 | 0.13, 3.77 | 0.7 |
| < 3cm | 64 (66.7) | 7 (7.3) |  |  |  |  |  |  |  |
| ≥ 3cm | 20 (20.8) | 5 (5.2) |  |  |  |  |  |  |  |
| Multifocality |  |  | 0.201 | 0.31 | 0.05, 1.27 | 0.15 | 0.18 | 0.02, 0.99 | 0.072 |
| no | 51 (53.1) | 10 (10.4) |  |  |  |  |  |  |  |
| yes | 33 (34.4) | 2 (2.1) |  |  |  |  |  |  |  |
| mpMRI |  |  | < 0.001 | 40.3 | 7.15, 762 | < 0.001 | 54.6 | 8.26, 1,134 | < 0.001 |
| VI-RADS ≤ 2 | 66 (68.8) | 1 (1.0) |  |  |  |  |  |  |  |
| VI-RADS ≥ 3 | 18 (18.8) | 11 (11.4) |  |  |  |  |  |  |  |
| *Unless otherwise indicated, data are number of patients and data in parentheses are percentages. CI = Confidence Interval, mpMRI = multiparametric magnetic resonance imaging OR = Odds Ratio, VI-RADS = Vesical Imaging-Reporting and Data System. | | | | | | | | | |
