# Supplementary material for: The role of VI-RADS scoring criteria for predicting oncological outcomes in bladder cancer
Source: World J Urol. 2024 Jul 24;42(1):438. doi: 10.1007/s00345-024-05101-2 (PMC11269435; doi:10.1007/s00345-024-05101-2)
Supplement: Supplementary file 4 — Supplementary Material 4 [file 345_2024_5101_MOESM4_ESM.docx]

| **Supplementary Information 4. Univariate and multivariable analysis of the impact of possible prognostic factors of recurrence and progression in the whole group.** | | | | | | | | |
| --- | --- | --- | --- | --- | --- | --- | --- | --- |
| Variables *N* (%) |  |  | univariate | | | multivariable | | |
|  | **Recurrence** | |  | | |  | | |
|  | **no** | **yes** | OR | 95% CI | *P* Value | OR | 95% CI | *P* Value |
| Multifocality |  |  | 2.37 | 0.94, 6.12 | 0.069 | 2.89 | 1.07, 8.12 | 0.038 |
| no | 37 (44.5) | 13 (15.7) |  |  |  |  |  |  |
| yes | 18 (21.7) | 15 (18.1) |  |  |  |  |  |  |
| Tumor diameter |  |  | 1.19 | 0.39, 3.43 | 0.7 | 1.35 | 0.37, 4.79 | 0.6 |
| ≥ 3cm | 43 (51.8) | 21 (24.3) |  |  |  |  |  |  |
| < 3cm | 12 (14.4) | 7 (8.4) |  |  |  |  |  |  |
| Prior recurrence |  |  | 1.99 | 0.72, 5.47 | 0.2 | 2.10 | 0.63, 7.09 | 0.2 |
| no | 43 (51.8) | 18 (21.7) |  |  |  |  |  |  |
| yes | 12 (14.5) | 10 (12.0) |  |  |  |  |  |  |
| T stage |  |  | 0.95 | 0.37, 2.51 | >0.9 | 0.55 | 0.14, 2.07 | 0.4 |
| Ta | 19 (22.8) | 10 (12.0) |  |  |  |  |  |  |
| T1 | 36 (43.5) | 18 (21.7) |  |  |  |  |  |  |
| Concurrent Cis |  |  | 2.00 | 0.08, 51.9 | 0.6 | NA | 0.00, NA | >0.9 |
| no | 54 (65.1) | 27 (32.5) |  |  |  |  |  |  |
| yes | 1 (1.2) | 1 (1.2) |  |  |  |  |  |  |
| Grade WHO 1973 |  |  | 1.75 | 0.07, 21.6 | 0.7 | 3.07 | 0.86, 12.6 | 0.10 |
| G1 | 21 (25.3) | 6 (7.3) |  |  |  |  |  |  |
| G2 | 32 (38.5) | 21 (25.3) |  |  |  |  |  |  |
| G3 | 2 (2.4) | 1 (1.2) |  |  |  |  |  |  |
| mpMRI |  |  | 1.80 | 0.61, 5.26 | 0.3 | 1.61 | 0.44, 5.79 | 0.5 |
| VI-RADS ≤ 2 | 45 (54.2) | 20 (24.1) |  |  |  |  |  |  |
| VI-RADS ≥ 3 | 10 (12.1) | 8 (9.6) |  |  |  |  |  |  |
|  | **Progression** | |  |  |  |  |  |  |
|  | **no** | **yes** |  |  |  |  |  |  |
| Age |  |  | 0.00 | NA, NA | >0.9 | NA | NA, NA | NA |
| ≤ 70 years | 46 (55.4) | 7 (8.4) |  |  |  |  |  |  |
| > 70 years | 30 (36.2) | 0 (0.0) |  |  |  |  |  |  |
| Prior recurrence |  |  | 4.30 | 0.87, 23.6 | 0.072 | 4.32 | 0.72, 31.1 | 0.11 |
| no | 58 (69.9) | 3 (3.6) |  |  |  |  |  |  |
| yes | 18 (21.7) | 4 (4.8) |  |  |  |  |  |  |
| Multifocality |  |  | 0.58 | 0.08, 2.88 | 0.5 | 0.67 | 0.09, 3.66 | 0.7 |
| no | 45 (54.2) | 5 (6.0) |  |  |  |  |  |  |
| yes | 31 (37.4) | 2 (2.4) |  |  |  |  |  |  |
| T stage |  |  | 0.37 | 0.07, 1.79 | 0.2 | 0.36 | 0.04, 3.17 | 0.3 |
| Ta | 25 (30.1) | 4 (4.8) |  |  |  |  |  |  |
| T1 | 51 (61.5) | 3 (3.6) |  |  |  |  |  |  |
| Grade WHO 1973 |  |  | 1.30 | 0.26, 9.54 | 0.8 | 1.95 | 0.24, 19.2 | 0.5 |
| G1 | 25 (30.1) | 2 (2.4) |  |  |  |  |  |  |
| G2 | 48 (57.9) | 5 (6.0) |  |  |  |  |  |  |
| G3 | 3 (3.6) | 0 (0.0) |  |  |  |  |  |  |
| Grade WHO 2004/2016 |  |  | 1.10 | 0.06, 7.44 | >0.9 | 2.32 | 0.09, 34.4 | 0.5 |
| low-grade | 66 (79.5) | 6 (7.2) |  |  |  |  |  |  |
| high-grade | 10 (12.1) | 1 (1.2) |  |  |  |  |  |  |
| Concurrent Cis |  |  | 0.00 | NA, NA | >0.9 | NA | NA, NA | NA |
| no | 74 (89.2) | 7 (8.4) |  |  |  |  |  |  |
| yes | 2 (2.4) | 0 (0.0) |  |  |  |  |  |  |
| mpMRI |  |  | 1.50 | 0.20, 7.70 | 0.6 | 2.00 | 0.21, 15.1 | 0.5 |
| VI-RADS ≤ 2 | 60 (72.3) | 5 (6.0) |  |  |  |  |  |  |
| VI-RADS ≥ 3 | 16 (19.3) | 2 (2.4) |  |  |  |  |  |  |
| *Unless otherwise indicated, data are number of patients and data in parentheses are percentages. CI = Confidence Interval, Cis = carcinoma in situ, mpMRI = multiparametric magnetic resonance imaging OR = Odds Ratio, VI-RADS = Vesical Imaging-Reporting and Data System, WHO = World Health Organisation. | | | | | | | | |
