# Supplementary material for: The role of VI-RADS scoring criteria for predicting oncological outcomes in bladder cancer
Source: World J Urol. 2024 Jul 24;42(1):438. doi: 10.1007/s00345-024-05101-2 (PMC11269435; doi:10.1007/s00345-024-05101-2)
Supplement: Supplementary file 5 — Supplementary Material 5 [file 345_2024_5101_MOESM5_ESM.docx]

| **Supplementary Information 5. Clinical characteristics and VI-RADS evaluation for the total group.** | | | | | |
| --- | --- | --- | --- | --- | --- |
| Variables n (%) | VI-RADS 1  n = 23 (24.0) | VI-RADS 2  n = 44 (45.8) | VI-RADS 3  n = 19 (19.8) | VI-RADS 4  n = 5 (5.2) | VI-RADS 5  n = 5 (5.2) |
| Age |  |  |  |  |  |
| ≤ 70 years | 21 (91.3) | 22 (50.0) | 13 (68.4) | 2 (40.0) | 2 (40.0) |
| > 70 years | 2 (8.7) | 22 (50.0) | 6 (31.6) | 3 (60.0) | 3 (60.0) |
| Multifocality |  |  |  |  |  |
| no | 15 (65.2) | 27 (61.4) | 12 (63.2) | 3 (60.0) | 4 (80.0) |
| yes | 8 (34.8) | 17 (38.6) | 7 (36.8) | 2 (40.0) | 1 (20.0) |
| Tumor diameter |  |  |  |  |  |
| ≥ 3cm | 0 (0.0) | 12 (27.3) | 9 (47.4) | 1 (20.0) | 3 (60.0) |
| < 3cm | 23 (100.0) | 32 (72.7) | 10 (52.6) | 4 (80.0) | 2 (40.0) |
| T stage |  |  |  |  |  |
| ≤ Ta | 15 (65.2) | 11 (25.0) | 4 (21.1) | 0 (0.0) | 0 (0.0) |
| T1a | 8 (34.8) | 30 (68.2) | 8 (42.1) | 0 (0.0) | 0 (0.0) |
| T1b | 0 (0.0) | 2 (4.5) | 2 (10.5) | 2 (40.0) | 0 (0.0) |
| ≥ T2 | 0 (0.0) | 1 (2.3) | 3 (15.8) | 3 (60.0) | 5 (100.0) |
| Cis | 0 (0.0) | 0 (0.0) | 2 (10.5) | 0 (0.0) | 0 (0.0) |
| Grade WHO 1973 |  |  |  |  |  |
| G1 | 11 (47.8) | 15 (34.1) | 2 (10.5) | 0 (0.0) | 0 (0.0) |
| G2 | 12 (52.2) | 29 (65.9) | 14 (73.7) | 3 (60.0) | 4 (80.0) |
| G3 | 0 (0.0) | 0 (0.0) | 3 (15.8) | 2 (40.0) | 1 (20.0) |
| Grade WHO 2004/2016 |  |  |  |  |  |
| low-grade | 23 (100.0) | 40 (90.9) | 10 (52.6) | 0 (0.0) | 0 (0.0) |
| high-grade | 0 (0.0) | 4 (9.1) | 9 (47.4) | 5 (100.0) | 5 (100.0) |
| *Unless otherwise indicated, data are number of patients and data in parentheses are percentages. VI-RADS = Vesical Imaging-Reporting and Data System, WHO = World Health Organisation. | | | | | |
